# Time-stratified HEART score discrimination and calibration in emergency chest pain: a retrospective cohort study

**DOI:** 10.1186/s12873-026-01652-2

**Published:** 2026-06-17

**Authors:** Şebnem Zeynep Eke Kurt, Uğur Durmuş, Erdem Kurt, Suphi Bahadırlı

**Affiliations:** 1 Department of Emergency Medicine, Taksim Training and Research Hospital, Istanbul, Turkey; 2 Department of Emergency Medicine, Istanbul Medeniyet University Göztepe Prof. Dr. Süleyman Yalçın Şehir Hastanesi, Istanbul, Turkey; 3 Department of Emergency Medicine, Istanbul Training and Research Hospital, Cerrahpaşa, Org. Abdurrahman Nafiz Gürman Cd. No:24, Fatih/Istanbul, 34098 Turkey; 4 Department of Emergency Medicine, Istanbul Medipol Mega University Hospital, Istanbul, Turkey

**Keywords:** Chest pain, Emergency service, Hospital, Risk assessment

## Abstract

**Aims:**

We compared HEART score discriminative performance and calibration for 30-day major adverse cardiac events (MACE) between off-hours and on-hours emergency department chest pain presentations.

**Methods:**

A single-center retrospective cohort study was conducted at Istanbul Medipol Mega University Hospital from January 2020 through December 2025, enrolling 2,800 patients (off-hours *n* = 2,133; on-hours *n* = 667). Off-hours comprised evenings, nights, weekends, and public holidays. The primary outcome was 30-day MACE, defined as acute myocardial infarction, unplanned coronary revascularization, cardiac arrest, or all-cause death. Discrimination was compared by the DeLong method; an absolute between-group AUC difference below 0.08, set a priori, was used as the margin of clinical similarity. Calibration was assessed by the Hosmer-Lemeshow test, observed-to-expected ratio, and calibration slope.

**Results:**

30-day MACE occurred in 14.4% of 2,800 patients. The HEART score AUC was 0.830 (95% CI 0.805–0.855) in off-hours and 0.821 (95% CI 0.778–0.865) in on-hours patients; ΔAUC was + 0.009 (95% CI -0.041 to + 0.058; *p* = 0.736), with the confidence interval contained within the a priori margin. At the rule-out threshold (HEART ≥ 4), off-hours sensitivity was 95.7% (95% CI 92.8–97.5%) and negative predictive value (NPV) was 97.7% (95% CI 96.2–98.7%). No differential shift effect was detected (interaction OR 1.039; 95% CI 0.867–1.247; *p* = 0.676). Both groups demonstrated systematic overestimation of absolute MACE risk relative to Backus 2013 probabilities (off-hours calibration intercept − 0.352; 95% CI -0.495 to -0.208; *p* < 0.001), with no between-group difference (*p* = 0.998).

**Conclusion:**

The HEART score showed clinically similar discrimination for 30-day MACE regardless of presentation time, supporting its use across different presentation times in tertiary-care settings with comparable infrastructure, pending prospective multicenter confirmation. Local recalibration of absolute risk estimates should be considered before probability-guided triage protocols are implemented in comparable settings.

**Supplementary Information:**

The online version contains supplementary material available at 10.1186/s12873-026-01652-2.

## Introduction

Acute chest pain accounts for approximately 5–10% of all adult emergency department visits. It is also one of the most demanding diagnostic problems in emergency medicine: about 10% of these patients receive a diagnosis of acute coronary syndrome, while most have non-cardiac causes [1]. Identifying that minority at triage, without subjecting every patient to prolonged observation or invasive workup, requires a standardized risk stratification instrument suitable for bedside use. The HEART score, integrating History, Electrocardiogram, Age, Risk Factors, and Troponin into a 10-point composite, was developed for this purpose [2] and subsequently validated in a multicenter retrospective cohort of 880 patients, demonstrating that scores of 0–3 were associated with a MACE rate below 1% [3]. Subsequent prospective validation across 2,440 patients in 10 Dutch emergency departments confirmed a C-statistic of 0.83, with 30-day MACE rates below 2% in the low-risk stratum and exceeding 50% in the high-risk stratum [3]. These risk thresholds have since anchored clinical disposition decisions in emergency departments across multiple countries [4]. 

External validation studies have confirmed HEART score discrimination in Asian emergency populations, with a C-statistic of 0.83 across 14 hospitals and 2,906 patients, and in retrospective military emergency cohorts, where AUCs of 0.885–0.898 were reported [5, 6]. A systematic review and meta-analysis incorporating 25 validation studies and more than 25,000 patients demonstrated a pooled sensitivity of 0.96 and negative predictive value of 0.99 for low-risk scores, confirming a consistent safety profile for rule-out [4]. However, to the best of our knowledge, no published validation study has stratified HEART score performance by time of emergency presentation. This gap is clinically relevant: off-hours admissions for acute coronary syndrome are associated with a small but persistent increase in in-hospital mortality, longer door-to-treatment intervals, and reduced specialist availability [7, 8]. Whether these operational differences translate into measurable differences in HEART score discriminative performance or calibration has not been formally examined in the published literature.

This study aimed to evaluate and compare the discriminative performance and calibration of the HEART score for 30-day MACE prediction in adult patients presenting with acute chest pain during off-hours versus standard working hours at a tertiary care emergency department. Secondary objectives included assessment of HEART score calibration relative to published validation cohort reference probabilities within each shift group, comparison of threshold-based rule-out and rule-in performance at the standard clinical cut points, evaluation of discrimination across five granular time strata, and decision curve analysis across a clinically relevant threshold probability range.

## Methods

### Study design and setting

This single-center retrospective cohort study was conducted in the emergency department of Istanbul Medipol Mega University Hospital, a tertiary care academic center in Istanbul, Turkey, between January 2020 and December 2025. The study constituted an external validation of the HEART score for 30-day MACE prediction and was classified as TRIPOD Type 2b/3. The study was approved by the Istanbul Medipol University Non-Interventional Clinical Research Ethics Committee (approval number: E-10840098-202.3.02-470; decision number: 90). Individual patient consent was waived in accordance with the retrospective design. A systematic search of PubMed and Embase (January 2000 through December 2024) using the terms ‘HEART score’, ‘off-hours’, ‘night shift’, ‘weekend’, and ‘time of presentation’ identified no prior study that stratified HEART score validation by time of emergency presentation, supporting the originality of the research question.

### Participants

Adult patients aged 18 years or older presenting to the emergency department with acute non-traumatic chest pain as the primary complaint were eligible for inclusion, provided that a first-visit triage timestamp was documented. Patients were excluded if HEART score documentation was incomplete (*n* = 298), if they presented with ST-elevation myocardial infarction requiring immediate primary percutaneous coronary intervention bypassing standard triage stratification (*n* = 1163), if they were transferred from another institution with an established cardiac diagnosis (*n* = 45), or if 30-day follow-up could not be ascertained (*n* = 167), yielding an analytic cohort of 2,800 patients from 4473 screened. The unit of analysis was the individual emergency presentation; the first-visit flag confirmed a first-presentation design with no repeated-measures clustering. The derivation of the analytic cohort from the screened population is illustrated in Figure S1.

### Variables

#### Primary exposure

The triage timestamp was used to classify each presentation as off-hours or on-hours. Off-hours encompassed evenings (18:00–23:59), nights (00:00–07:59), weekends (Saturday and Sunday, all hours), and public holidays. On-hours encompassed regular weekday working hours (08:00–17:59, Monday through Friday, excluding public holidays). Under this definition, off-hours encompassed approximately 75% of total weekly hours, reflecting the predominance of evenings, nights, weekends, and public holidays in the calendar; the resulting 76.2% off-hours enrollment fraction is therefore consistent with the expected distribution of presentations across this time window.

#### Index test

The HEART score was calculated from five components, each scored 0–2: History, Electrocardiogram, Age, Risk Factors, and Troponin, yielding a total score of 0–10 [2]. Patients were classified as low risk (score 0–3), intermediate risk (score 4–6), or high risk (score 7–10). Predicted probabilities for calibration analyses were derived from the Backus 2013 prospective validation cohort reference values (hereafter “the reference cohort”), low-risk group 0.017, intermediate-risk group 0.120, and high-risk group 0.652 [9]. Logit-scale values of these probabilities were pre-computed for offset regression analyses.

#### Primary outcome

The primary outcome was 30-day MACE, defined as a composite of acute myocardial infarction, unplanned coronary revascularization, cardiac arrest, or all-cause death occurring within 30 days of the index emergency presentation, ascertained through hospital readmission records, cardiology outpatient records, and institutional mortality registries. Elective revascularization procedures were excluded from the composite endpoint.

#### Covariates

The first troponin measurement (Troponin-1, ng/L) was recorded at triage. Door-to-diagnosis time was defined as the interval in minutes from triage timestamp to documented clinical diagnosis. Troponin assay type (high-sensitivity troponin I versus conventional troponin I) and ECG interpreter seniority (resident, specialist, or cardiologist) were recorded as secondary variables. Comorbidities (hypertension, diabetes mellitus, hyperlipidemia, current smoking, obesity) were ascertained from the electronic medical record.

### Data sources and measurement

Clinical data were extracted from the institutional electronic medical record system using a standardized data collection protocol. HEART score components were documented by the treating emergency physician at the time of presentation and abstracted verbatim from the medical record. The troponin assay transitioned from conventional troponin I to high-sensitivity troponin I during the study period; assay type was recorded per patient and addressed in a sensitivity analysis. ECG interpretation was performed by the treating clinician and the interpreter’s seniority level was recorded. The 30-day follow-up was ascertained from institutional records without active patient contact.

### Bias

Four principal sources of bias were addressed prospectively. Information bias from retrospective record abstraction was minimized by a standardized extraction protocol applied uniformly across both shift groups. Residual observer-dependent variability in the History component cannot be fully quantified; its expected direction of effect is non-differential between groups. Temporal confounding from the mid-study transition between troponin assay platforms affects the Troponin component of the HEART score and was addressed in Sensitivity Analysis 2 (SA2), which stratified the primary discrimination and calibration analyses by assay type; overlapping confidence intervals across assay subgroups indicate that this transition did not materially bias the primary estimate. Differential serial troponin completion between shift groups (off-hours 78.8%, on-hours 91.3%) is the most consequential source of potential outcome misclassification. SA1 restricted the primary analysis to patients with complete serial troponin measurements to characterize the direction and magnitude of this effect. A fourth source is outcome ascertainment bias from using institutional records without active patient contact. Patients who experienced MACE at external facilities would not be captured, which would bias the observed event rate downward and the negative predictive value upward; the magnitude cannot be quantified from the available data.

### Study size

As this was a retrospective study, no prospective power calculation was performed. All patients meeting eligibility criteria during the study period were included. The final analytic cohort comprised 2,800 patients, of whom 402 experienced the primary outcome (30-day MACE rate 14.4%). The off-hours group included 2,133 patients with 305 events and the on-hours group included 667 patients with 97 events. For the primary secondary estimand, the DeLong confidence interval for the AUC difference spanned − 0.041 to + 0.058, confirming adequate precision for the ± 0.08 margin of clinical similarity.

### Statistical analysis

#### Normality assessment

All primary continuous variables (age, HEART score, troponin-1, and door-to-diagnosis time) were non-normally distributed; the multi-criterion procedure used to establish this is described in the Supplement (Supplementary Methods 1).

#### Descriptive statistics and group comparisons

Continuous variables are reported as median (interquartile range) and compared between groups using the Mann-Whitney U test. Categorical variables are reported as n (%) and compared using the chi-square test with continuity correction for 2 × 2 tables or Fisher’s exact test where any expected cell count was below 5. For the three-level HEART risk group variable, a 3 × 2 chi-square test was applied with the global p-value reported. Standardized mean differences (SMD) were computed for all Table 1 variables as a p-value-independent measure of group balance; an SMD exceeding 0.10 was defined a priori as indicating clinically meaningful imbalance.

**Table 1:** Baseline characteristics by shift group

Variable	Off-hours (*n* = 2,133)	On-hours (*n* = 667)	*p*	SMD
**Continuous variables**				
Age, median (IQR), years	59 (49–70)	59 (49–67)	0.067	0.089
HEART total score, median (IQR)	5 (3–6)	4 (3–6)	0.604	0.014
Troponin-1, median (IQR), ng/L	18.4 (7.5–54.6)	17.7 (6.6–48.4)	0.345	0.024
Door-to-diagnosis time, median (IQR), min	168 (127–202)	146 (104–184)	< 0.001	0.351*
**Categorical variables**				
Male sex, n (%)	1,220 (57.2)	380 (57.0)	0.954	0.005
MACE at 30 days, n (%)	305 (14.3)	97 (14.5)	0.926	0.007
Acute myocardial infarction	228 (10.7)	74 (11.1)	0.824	0.013
Revascularisation	157 (7.4)	45 (6.7)	0.653	0.024
Cardiac arrest	38 (1.8)	7 (1.0)	0.256	0.062
Death	39 (1.8)	13 (1.9)	0.970	0.009
MACE at 6 weeks, n (%)	332 (15.6)	104 (15.6)	1.000	0.001
HEART risk group, n (%)			0.301	0.024
Low risk (score 0–3)	574 (26.9)	196 (29.4)		
Intermediate risk (score 4–6)	1,223 (57.3)	360 (54.0)		
High risk (score 7–10)	336 (15.8)	111 (16.6)		
**Comorbidities**				
Hypertension	960 (45.0)	275 (41.2)	0.095	0.076
Diabetes mellitus	513 (24.1)	161 (24.1)	1.000	0.002
Hyperlipidaemia	826 (38.7)	262 (39.3)	0.833	0.011
Current smoking	559 (26.2)	205 (30.7)	0.025	0.100*
Obesity	539 (25.3)	150 (22.5)	0.160	0.065
**Process indicators**				
Original serial troponin complete, n (%)	1,681 (78.8)	609 (91.3)	< 0.001	0.356*
hsTnI assay used, n (%)	1,458 (68.4)	458 (68.7)	0.918	0.007
Hospitalisation, n (%)	534 (25.0)	170 (25.5)	0.854	0.010
Cardiology consultation, n (%)	606 (28.4)	184 (27.6)	0.716	0.018

IQR = interquartile range; MACE = major adverse cardiac event; hsTnI = high-sensitivity troponin I; SMD = standardised mean difference. Continuous variables were compared by the Mann-Whitney U test and categorical variables by the chi-square test, with Fisher’s exact test where any expected cell count was below 5. SMD > 0.10 (marked *) indicates clinically meaningful imbalance. Individual MACE components are not mutually exclusive; a single patient may contribute to more than one component

#### Discrimination

The area under the receiver operating characteristic curve (AUC, equivalent to the C-statistic) was computed using the DeLong method, which provides exact confidence intervals without bootstrap approximation. Threshold-based performance metrics (sensitivity, specificity, positive predictive value, negative predictive value, positive likelihood ratio, negative likelihood ratio) were computed at two pre-specified clinical thresholds: HEART ≥ 4 (standard rule-out threshold) and HEART ≥ 7 (high-risk admission threshold). Wilson score confidence intervals were applied to all proportion-based metrics. The Youden index (J = sensitivity + specificity − 1) was computed across all score values to identify a data-driven optimal cutpoint; this cutpoint was exploratory and was not part of the primary analysis plan.

#### Calibration

Calibration was assessed using five complementary metrics: [1] the Hosmer-Lemeshow goodness-of-fit test, with one group per HEART score value (11 groups, score 0 through 10; df = 9 under the H-L formula df = g − 2); [2] the observed-to-expected (O/E) ratio overall and within each risk stratum, with Byar approximation for 95% confidence intervals; [3] the calibration intercept, estimated by offset logistic regression with the logit of reference cohort predicted probabilities as a fixed offset term [4] the calibration slope, estimated by logistic regression of the observed outcome on the logit of predicted probabilities, with bootstrap confidence intervals (1000 iterations; seed = 2024) and a two-sided z-test against the null hypothesis of slope = 1; and [5] the Brier score and scaled Brier score, quantifying overall predictive skill relative to the null model.

#### Decision curve analysis

Net benefit was computed across a threshold probability range of 5–40% and compared against treat-all and treat-none reference strategies. Separate decision curves were produced for off-hours and on-hours groups.

#### Primary comparative analysis

The primary secondary estimand was the difference in AUC between independent off-hours and on-hours groups, computed using the DeLong variance estimator for independent samples. Clinical similarity was defined a priori in the analysis plan as an absolute difference in AUC below 0.08. This margin was a pragmatic choice rather than a value derived from an external empirical standard: an equivalence or non-inferiority margin should represent a difference small enough to be clinically unimportant and should be justified on clinical and statistical grounds [10]. A difference in C-statistic below 0.08 was judged unlikely to change the rule-out and rule-in disposition decisions the HEART score is used to support at the standard thresholds. The comparison is therefore interpreted descriptively, with the confidence interval for the AUC difference as the primary basis for inference rather than a formal equivalence hypothesis test. A bootstrap confidence interval for ΔAUC was generated as a supplementary check (1,000 iterations; seed = 2024; percentile method). A formal interaction test was conducted using logistic regression including the mean-centered HEART score, off-hours status, and their product term; the interaction p-value served as the test of differential discrimination between groups.Within each HEART risk group, 30-day MACE rates were compared between shift groups using chi-square with continuity correction; the Mantel-Haenszel pooled odds ratio was computed with HEART risk group as the stratification variable.

#### Time strata analysis

Five-time strata were defined: on-hours (reference), evening, night, weekend daytime, and public holidays. An AUC was estimated within each stratum using the DeLong method. Heterogeneity across strata was assessed using a weighted Cochran Q statistic; pairwise comparisons against the on-hours reference were planned only in the event of a significant global test (*p* < 0.05), applying Benjamini-Hochberg false discovery rate correction.

#### Sensitivity analyses

Five sensitivity analyses were specified a priori. SA1 restricted the analysis to patients with complete serial troponin documentation, isolating the potential bias introduced by incomplete serial troponin measurements. SA2 stratified the off-hours cohort by troponin assay type (high-sensitivity troponin I versus conventional troponin I) and repeated the primary discrimination and calibration analyses within each subgroup, addressing the temporal confounding effect of assay migration on the T-component of HEART. SA3 stratified by the seniority of the clinician who interpreted the ECG (resident, specialist, or cardiologist), testing whether off-hours degradation in ECG interpretation quality affects E-component scoring and downstream model performance. SA4 replaced the 30-day MACE endpoint with the 6-week MACE outcome, aligning the follow-up window with the reference cohort and testing sensitivity of findings to outcome ascertainment duration. SA5 used MICE-imputed HEART scores in place of original scores (20 imputation sets, 50 iterations, seed = 2024, Rubin’s rules applied); missing data were confined to components with original documentation gaps, and no outcome imputation was performed. Full results for all sensitivity analyses are presented in Supplementary Table S1.

Two additional analyses used balanced alternative time definitions to assess sensitivity of the primary comparison to the off-hours definition: a calendar-based weekday-versus-weekend contrast, and a clock-based daytime (08:00–17:59) versus nighttime (18:00–07:59) contrast. Both were post hoc and are reported as exploratory.

All analyses were conducted in Python 3.11 (scipy 1.11+, statsmodels 0.14+, scikit-learn 1.3+). Statistical significance was set at *p* < 0.05 (two-tailed) for the primary outcome. All point estimates are accompanied by 95% confidence intervals.

### Missing data

Primary analysis variables (HEART total score, 30-day MACE, first troponin, and reference cohort predicted probabilities) contained no missing values. A complete-case flag identified 2,080 of 2,800 patients (74.3%) with complete data across all secondary variables. Patients with incomplete secondary variable data were excluded from the specific analyses requiring those variables; denominators are reported explicitly for each analysis step. Multiple imputation (SA5) was applied only to missing HEART score components, with imputed risk-group assignments used to derive predicted probabilities for that analysis alone. Serial troponin values were never imputed; patients had either complete or incomplete serial troponin documentation, and SA1 addressed this by restriction. The primary HEART total score and 30-day MACE outcome contained no imputed values. The 720 incomplete cases were driven by three variables: original serial troponin documentation (*n* = 510; 18.2%), family history (*n* = 180; 6.4%), and original ECG interpreter records (*n* = 86; 3.1%). Serial troponin missingness was differential by shift (off-hours 21.2% versus on-hours 8.7%), consistent with missing at random conditional on shift assignment and directly addressed by SA1; family history missingness was non-differential across shift groups (6.4% versus 6.6%), consistent with missing completely at random. ECG interpreter seniority was missing for 86 patients (3.1%), all within the on-hours group; documentation was complete for every off-hours patient. Because SA3 was restricted to the off-hours cohort, its subgroup counts are unaffected by this missingness and sum to the full off-hours sample (*n* = 2,133).

### Outcomes

The primary outcome was 30-day MACE, defined as a composite of acute myocardial infarction, unplanned coronary revascularization, cardiac arrest, or all-cause death within 30 days of the index presentation, specified a priori in the analysis plan. Secondary outcomes included MACE at 6 weeks, individual MACE components (reported separately), and HEART score discrimination stratified by time stratum. The primary secondary estimand was the difference in AUC between off-hours and on-hours groups. The primary and secondary estimands and the margin of clinical similarity were specified a priori in the analysis plan. The Youden-index cutpoint was a data-driven, exploratory addition that was not part of this plan; all confirmatory conclusions rest on the a priori estimands.

## Results

### Study cohort

The analytic cohort comprised 2,800 patients presenting with acute chest pain (Figure S1), of whom 2,133 (76.2%) presented during off-hours and 667 (23.8%) during on hours. Overall, 402 patients experienced a 30-day MACE (14.4%). All entries in the dataset carried a first-visit flag, confirming the absence of repeated-measure clustering.

### Baseline characteristics

Table 1 presents baseline characteristics by off-hours and on-hours status. The two groups were well-matched on age (both median 59 years), MACE rate (14.3% versus 14.5%, *p* = 0.926), sex distribution (57.2% versus 57.0% male), HEART score (median 5 versus 4, *p* = 0.604), first troponin (18.4 versus 17.7 ng/L, *p* = 0.345), and comorbidity profiles. Risk group distribution was also comparable (chi-square *p* = 0.301). Assay type was balanced: hsTnI was used in 68.4% of off-hours and 68.7% of on-hours patients (*p* = 0.918).

Three variables showed clinically meaningful imbalance (SMD > 0.10). Door-to-diagnosis time was longer in off-hours patients (median 168 versus 146 min, *p* < 0.001, SMD = 0.351), reflecting reduced specialist availability outside working hours. Serial troponin completion was lower in off-hours patients (78.8% versus 91.3%, *p* < 0.001, SMD = 0.356). Smoking prevalence also showed clinically meaningful imbalance (26.2% versus 30.7%, *p* = 0.025, SMD = 0.100). These imbalances are addressed in the relevant sensitivity analyses.

### Primary validation in the off-hours cohort

#### Discrimination

**Fig. 1:**
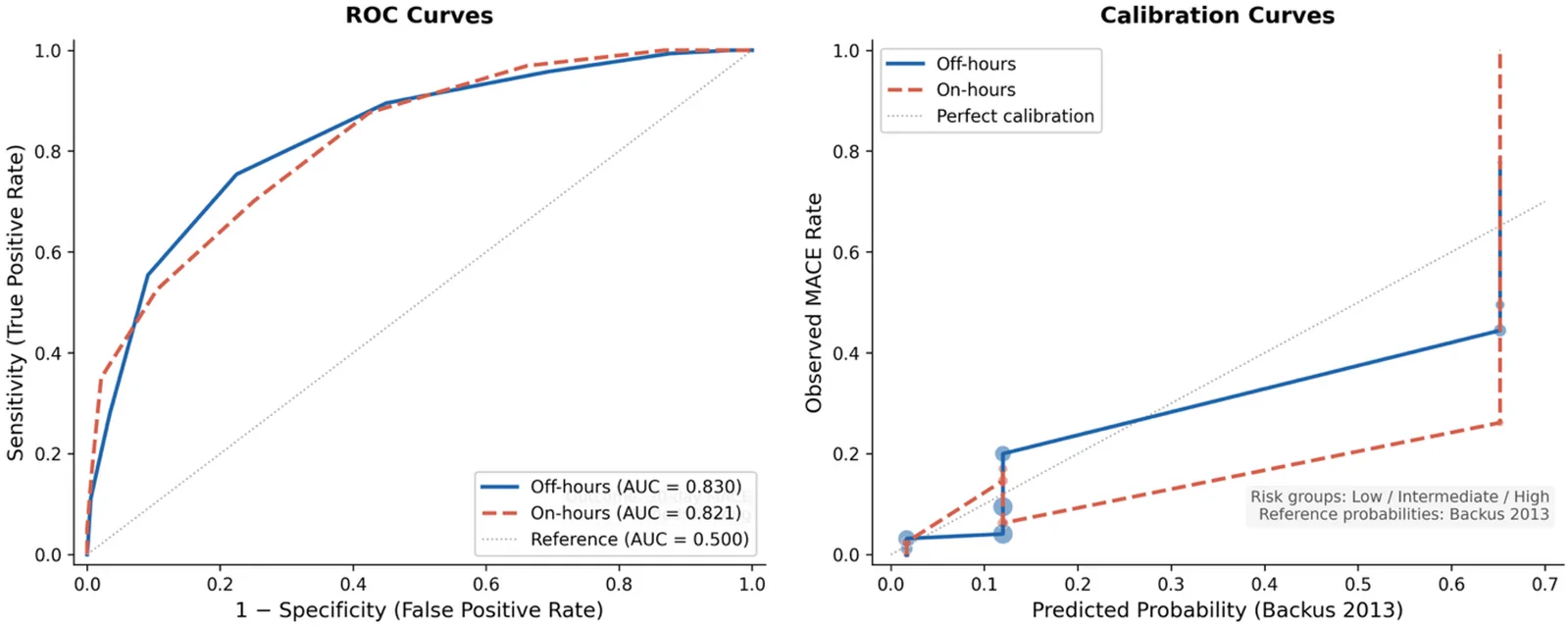
ROC curves and calibration plots for the HEART score against 30-day MACE. Left: Receiver operating characteristic curves for off-hours (*n* = 2,133; solid blue) and on-hours (*n* = 667; dashed red) groups. AUC estimated by DeLong method. Right: Calibration curves of observed versus predicted MACE probability by HEART score group; bubble size proportional to group size; dashed diagonal = perfect calibration; points below the diagonal indicate that the reference model assigns higher predicted risk than is observed. Predicted probabilities from the reference cohort. Both groups demonstrate systematic overestimation of absolute risk (O/E < 1). MACE = major adverse cardiac event; AUC = area under the ROC curve

In the off-hours group (*n* = 2,133; 305 MACE events, 14.3%), the HEART score achieved an AUC of 0.830 (95% CI: 0.805–0.855), confirming good discriminative performance (Fig. 1, left panel; Table 2).

**Table 2:** Discrimination metrics at pre-specified and exploratory thresholds (off-hours cohort, *n* = 2,133)

Metric	HEART ≥ 4 (standard)	HEART ≥ 7 (high-risk)	HEART ≥ 6 (Youden, exploratory)	95% CI method
AUC (C-statistic)				0.830 (0.805–0.855)
Sensitivity	95.7% (92.8–97.5)	55.4% (49.8–60.9)	75.4% (70.3–79.9)	Wilson
Specificity	30.7% (28.6–32.8)	90.9% (89.5–92.1)	77.5% (75.5–79.4)	Wilson
PPV	18.7% (16.9–20.7)	50.3% (45.0-55.6)	35.9% (32.3–39.7)	Wilson
NPV	97.7% (96.2–98.7)	92.4% (91.1–93.6)	95.0% (93.7–96.0)	Wilson
LR+	1.38	6.07	3.35	
LR-	0.139	0.491	0.317	
TP / FP / TN / FN	292/1267/561/13	169/167/1661/136	230/411/1417/75	

N = 2,133 off-hours patients; 305 MACE events (14.3%). Threshold ≥ 4: standard rule-out; threshold ≥ 7: high-risk rule-in; threshold ≥ 6: Youden-index optimal (post hoc, data-driven; exploratory, not part of the primary analysis plan). AUC computed by the DeLong method; proportion-based metrics with Wilson 95% CI. AUC = area under the ROC curve; CI = confidence interval; PPV = positive predictive value; NPV = negative predictive value; LR = likelihood ratio; TP/FP/TN/FN = true/false positive/negative. Other abbreviations as in Table 1

At the standard clinical rule-out threshold (HEART ≥ 4), sensitivity was 95.7% (95% CI: 92.8–97.5%) and NPV was 97.7% (95% CI: 96.2–98.7%), with 13 missed events among 574 patients below the threshold. At the high-risk admission threshold (HEART ≥ 7), specificity was 90.9% (95% CI: 89.5–92.1%) and PPV was 50.3% (95% CI: 45.0-55.6%). The positive likelihood ratio at this threshold was 6.07, indicating a clinically meaningful shift in post-test probability.

The Youden-index optimal threshold was HEART ≥ 6 (J = 0.529), with sensitivity 75.4% (95% CI: 70.3–79.9%) and specificity 77.5% (95% CI: 75.5–79.4%). This threshold was identified post hoc and is reported as exploratory.

#### Calibration

Table 3 presents the complete calibration profile. The Hosmer-Lemeshow test was highly significant (χ²=104.32, df = 9, *p* < 0.001), indicating systematic departure from the reference cohort probabilities. The overall O/E ratio of 0.812 indicates that the Backus model predicted approximately 23% more MACE events (predicted *N* = 375.6) than were observed (*N* = 305). This pattern of overestimation was consistent across all three risk strata, with O/E values of 1.33 in the low-risk group (confidence interval crossing 1.0, reflecting sparse events), 0.84 in the intermediate-risk group, and 0.77 in the high-risk group.

**Table 3:** Calibration metrics for off-hours and on-hours groups

Calibration metric	Off-hours (*n* = 2,133)	On-hours (*n* = 667)	Difference	*p*
Hosmer-Lemeshow χ² (df = 9)	104.32	16.83		< 0.001 / 0.052
Hosmer-Lemeshow p-value	< 0.001	0.052		
O/E ratio, overall (Byar 95% CI)	0.812 (0.724–0.908)	0.791 (0.642–0.965)		
Low-risk group (HEART 0–3)	1.332 (0.665–2.278)	0.625 (0.121–1.821)		
Intermediate-risk group (HEART 4–6)	0.838 (0.693-1.000)	0.860 (0.629–1.141)		
High-risk group (HEART 7–10)	0.771 (0.657–0.897)	0.747 (0.560–0.977)		
Calibration intercept (95% CI)	-0.352 (-0.495 to -0.208)	-0.351 (-0.608 to -0.094)	-0.000	0.998
Calibration slope (bootstrap 95% CI)	0.828 (0.732–0.916)	0.758 (0.624–0.910)	0.070	0.426
Brier score	0.101	0.109		
Scaled Brier score	0.176	0.125		

A Hosmer-Lemeshow *p* < 0.001 indicates systematic miscalibration relative to reference-cohort probabilities; O/E < 1 indicates the model overestimates absolute MACE risk in this population. The calibration intercept tested H₀: intercept = 0 and the slope tested H₀: slope = 1; intercept and slope differences used a z-test with bootstrap standard errors, and all other metrics are descriptive. O/E = observed-to-expected ratio. Other abbreviations as in Tables 1 and 2

The calibration intercept was − 0.352 (95% CI: -0.495 to -0.208; *p* < 0.001), indicating that the reference model systematically overestimates absolute MACE risk in this cohort, consistent with a lower baseline event rate than the reference population. The calibration slope was 0.828 (bootstrap 95% CI: 0.732–0.916; *p* < 0.001 for deviation from 1.0), indicating miscalibration in the extremes: the reference model overestimates risk in higher-scoring patients and underestimates it in lower-scoring patients relative to what is observed in this cohort. The calibration curve (Fig. 1, right panel) illustrates both the downward shift and the reduced steepness of observed versus predicted risk across score values.

The Brier score was 0.101 and the scaled Brier score was 0.176, reflecting meaningful predictive skill above the null (scaled Brier 0 = no skill; 1 = perfect). The significant intercept deviation suggests that local recalibration of absolute risk estimates may be warranted, pending prospective confirmation.

#### Decision curve analysis

**Fig. 2:**
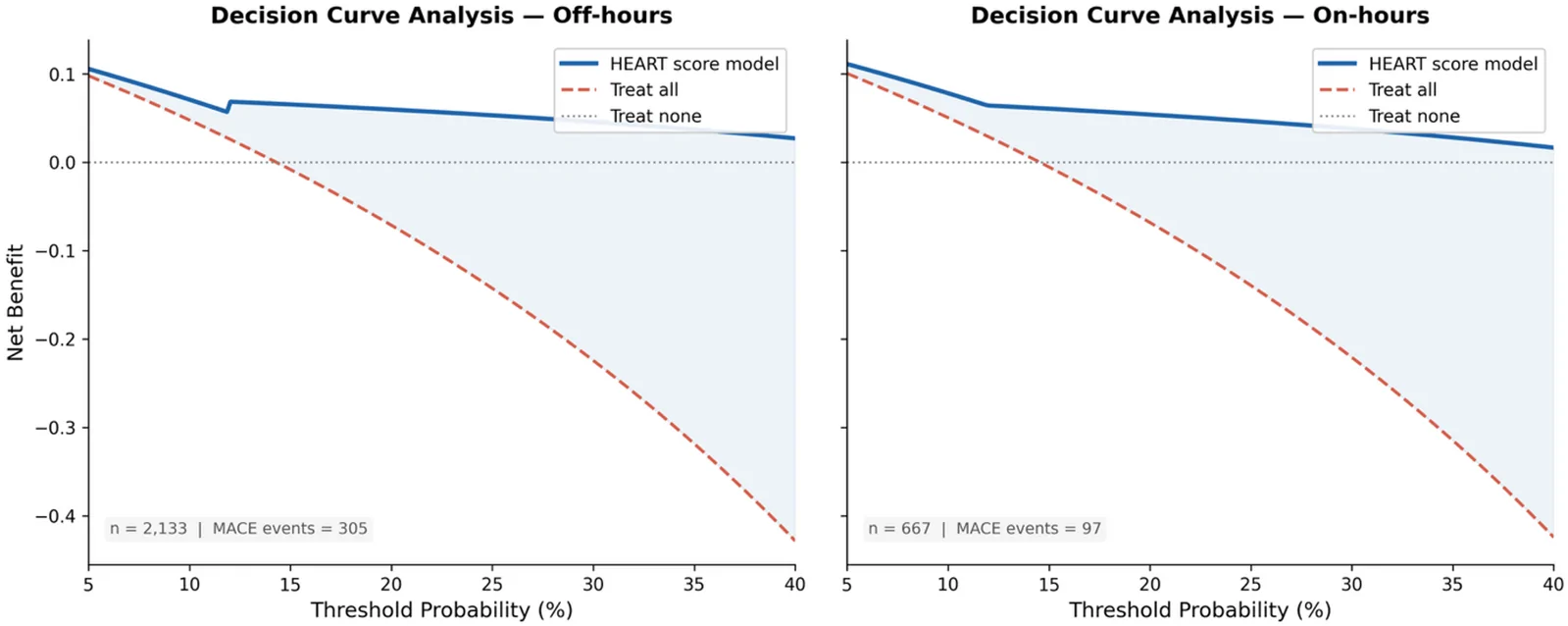
Decision curves for the HEART score model compared with treat-all and treat-none reference strategies. Left: Off-hours group (*n* = 2,133). Right: On-hours group (*n* = 667). X-axis: clinician’s threshold probability for treatment (hospitalisation/further workup); Y-axis: net benefit per patient evaluated. The HEART score model provides positive net benefit over both reference strategies across the threshold range (5–40%). DCA = decision curve analysis

Figure 2 shows decision curves for off-hours and on-hours groups across threshold probabilities of 5–40%. The HEART score model delivered positive net benefit exceeding both the treat-all and treat-none strategies across the full threshold range in both groups. At a threshold of 10%, net benefit was 0.071 in off-hours patients versus a treat-all net benefit of 0.048, a net advantage of 0.023 per patient evaluated, equivalent to avoiding approximately 23 unnecessary hospitalizations per 1,000 patients assessed. Unlike the AUC, which summarizes ranking ability irrespective of any decision threshold, the decision curve quantifies the net clinical consequence of acting on the score at specific thresholds; it shows that applying the HEART score yields more benefit than treating all or no patients across the plausible decision range, with no evidence of differential performance between shift groups.

### Comparative analysis

#### AUC comparison between groups

The primary secondary estimand, the difference in AUC between off-hours and on-hours groups, was ΔAUC = + 0.009 (95% CI: -0.041 to + 0.058; *p* = 0.736) in favour of off-hours patients (Table 4). The bootstrap-derived 95% CI was − 0.040 to + 0.057. Both the point estimate and the entire confidence interval fell well within the ± 0.08 margin of clinical similarity, providing clear evidence that HEART score discrimination does not meaningfully differ by shift assignment. The AUC in the on-hours group was 0.821 (95% CI: 0.778–0.865), consistent with the off-hours estimate.

**Table 4:** AUC comparison between off-hours and on-hours groups

Analysis	Off-hours AUC (95% CI)	On-hours AUC (95% CI)	ΔAUC (95% CI)	*p*
Primary (30-day MACE)	0.830 (0.805–0.855)	0.821 (0.778–0.865)	+ 0.009 (-0.041 to + 0.058)	0.736
Bootstrap ΔAUC (1,000 iterations; seed = 2024)			(-0.040 to + 0.057)	
6-week MACE (SA4)	0.818 (0.793–0.843)	0.811 (0.767–0.855)	+ 0.007 (-0.044 to + 0.058)	0.790

ΔAUC = off-hours minus on-hours; positive values favour off-hours. DeLong method (independent groups) for the primary comparison. Margin of clinical similarity: ΔAUC < 0.08, specified a priori in the analysis plan. Abbreviations as in Tables 1 and 2

#### Interaction analysis

The formal interaction model confirmed the absence of differential HEART score effects by shift. The interaction term (mean-centred HEART score × off-hours indicator) had OR = 1.039 (95% CI: 0.867–1.247; *p* = 0.676), providing no evidence that the HEART score’s log-odds slope for MACE differs between off-hours and on-hours patients. The main effect of the HEART score was OR = 2.166 per unit increase (95% CI: 1.851–2.533; *p* < 0.001), consistent with the overall discriminative performance. The off-hours main effect was OR = 0.955 (95% CI: 0.655–1.393; *p* = 0.811), confirming no independent effect of shift on MACE risk after accounting for HEART score.

#### MACE rates by risk group

Supplementary Table S3 presents 30-day MACE rates within each HEART risk group by shift. Rates were 2.3% versus 1.5% (*p* = 0.740) in the low-risk group, 10.1% versus 11.9% (*p* = 0.353) in the intermediate-risk group, and 50.3% versus 45.9% (*p* = 0.493) in the high-risk group. No statistically significant difference was observed in any stratum. The Mantel-Haenszel pooled OR, adjusted for HEART risk group, was 0.989 (95% CI: 0.752–1.302; *p* = 0.995), confirming no significant difference in MACE risk between off-hours and on-hours patients across strata. MACE component breakdown by risk group and time stratum is shown in Supplementary Figure S2.

#### Time strata analysis

Supplementary Table S2 presents AUC estimates across the five time strata. AUC ranged from 0.799 (95% CI: 0.747–0.850) in weekend daytime presentations to 0.855 (95% CI: 0.810–0.899) in evening presentations. The Cochran Q statistic was 2.991 (df = 4, *p* = 0.559), providing no evidence of significant heterogeneity across strata. Per the pre-specified decision rule, pairwise comparisons were not performed. The HEART score maintained good discrimination across all time categories including public holidays (AUC = 0.835, 95% CI: 0.779–0.891).

#### Calibration comparison

Calibration metrics were statistically indistinguishable between groups. The intercept difference was − 0.0003 (bootstrap 95% CI: -0.350 to + 0.327; *p* = 0.998), and the slope difference was 0.070 (*p* = 0.426). Both groups exhibited comparable systematic miscalibration relative to Backus 2013, with significant intercept deviations (off-hours *p* < 0.001; on-hours *p* = 0.007) and significant slope departures from 1.0 (both *p* < 0.001). This indicates that the recalibration need identified in the primary analysis applies equally to both shift groups.

#### Balanced alternative time definitions

Under balanced alternative time definitions, HEART score discrimination did not differ significantly between groups (Supplementary Table S4). Discrimination was similar for weekday (AUC 0.839; 95% CI 0.813–0.864) versus weekend presentations (AUC 0.803; 95% CI 0.764–0.843; ΔAUC − 0.035; 95% CI -0.082 to + 0.012; *p* = 0.142) and for daytime (AUC 0.810; 95% CI 0.776–0.844) versus nighttime presentations (AUC 0.843; 95% CI 0.816–0.869; ΔAUC − 0.032; 95% CI -0.076 to + 0.011; *p* = 0.145). Both point estimates fell within the a priori margin of 0.08; the weekend comparison’s confidence interval extended marginally beyond the lower margin bound, consistent with reduced precision in the smaller weekend subgroup.

### Sensitivity analyses

Supplementary Table S1 and Supplementary Figure S3 present the sensitivity analyses. Across all scenarios, AUC estimates in the off-hours group ranged from 0.818 (SA4, 6-week endpoint) to 0.848 (SA2b, conventional troponin assay subgroup), with the primary estimate of 0.830 consistent with all sensitivity results.

In SA1 (original serial troponin complete; *n* = 1,681), AUC was 0.841 (95% CI: 0.815–0.867), marginally higher than the primary estimate, with a calibration intercept of -0.294 (versus − 0.352 in the full cohort). The modest improvement suggests that the 21.2% of patients with incomplete serial troponin measurements did not introduce systematic bias, although their inclusion slightly attenuated discrimination.

SA2 showed consistent performance across assay types: AUC was 0.821 (95% CI: 0.791–0.851) with hsTnI and 0.848 (95% CI: 0.804–0.892) with conventional troponin, with overlapping confidence intervals. SA3 demonstrated stable HEART score performance regardless of ECG interpreter seniority (AUC range 0.824–0.833), with overlapping confidence intervals across resident, specialist, and cardiologist groups. SA5 with MICE-imputed scores yielded AUC = 0.835, marginally above the primary estimate, consistent with expected slight optimism correction from imputation.

## Discussion

This study found that HEART score discrimination for 30-day MACE was clinically similar across all hours of emergency presentation, with the AUC difference between shift groups falling well within the margin of clinical similarity and a formal interaction test confirming no differential score effect by shift assignment. These findings indicate that the operational constraints characteristic of off-hours care, including longer door-to-diagnosis intervals and lower serial troponin completion rates, did not materially impair the score’s ability to classify patients by MACE risk.

The HEART score integrates five independently scored components, no single one of which dominates the composite discriminative gradient. Components anchored to fixed patient characteristics, specifically Age and Risk Factors, remain unaffected by shift-related workflow variation. The History and ECG components are the most subjectively scored elements: a prospective comparison of clinician-calculated and researcher-generated HEART scores found overall agreement of 78% (kappa 0.48), with the History component achieving the lowest pairwise kappa of 0.14 and ECG kappa of 0.40 [11]. Despite this component-level variability, inter-rater reliability among emergency physicians, who staff departments continuously across shifts, was shown to be substantial for low-risk classification (kappa 0.68; 84.2% agreement) [12]. The composite score architecture may therefore tolerate component-level variability without proportional degradation of aggregate discriminative performance across shift conditions.

The AUC of 0.830 in the off-hours cohort aligns with the Backus 2013 prospective multicenter validation cohort (AUC 0.83 across 10 Dutch centers) and the Six 2013 multinational Asia-Pacific validation of 2,906 patients (AUC 0.83) [5, 9]. A systematic review and meta-analysis of 25 validation studies and more than 25,000 patients reported a pooled sensitivity of 0.96 and NPV of 0.99 for low-risk scores, with a central C-statistic of approximately 0.83 across heterogeneous institutional contexts [4]. In a direct comparison among 1,748 chest pain patients across nine Dutch hospitals, the HEART score outperformed the GRACE score (AUC 0.73) and TIMI score (AUC 0.80), identifying 381 low-risk patients with only 0.8% missed MACE compared with 2.2% under GRACE at equivalent safety levels [13]. Higher AUC values have been reported in a US military emergency cohort (0.885) [6], in institutions operating under formal HEART pathway protocols (0.898) [14], and in a recent Middle Eastern tertiary validation where baseline MACE prevalence was 24.8% (AUC 0.925) [15]. In contrast, a prospective Tanzanian cohort of 927 patients yielded an AUC of only 0.61, with sensitivity of 59.4% and NPV of 74.7% at the ≥ 4 threshold, attributed to limited ECG interpretation infrastructure and absence of high-sensitivity troponin assays [16]. This low-income context finding indicates that HEART score discrimination is not uniformly high across settings; the reported AUC range of 0.61 to 0.925 appears to track assay quality and clinical interpretation infrastructure more closely than time of presentation. The present single-center estimate should be read as setting-specific rather than as evidence of uniform performance. Weekend effect meta-analyses in ACS consistently show higher in-hospital mortality among weekend versus weekday admissions (pooled OR 1.06; 95% CI 1.03–1.09 across 18 studies and more than 14 million ACS patients) [7]. A Spanish national registry analysis reported 5% higher STEMI mortality and 8% higher NSTEACS mortality on weekends and public holidays after risk adjustment [8]. These outcome data are drawn from acute coronary syndrome systems-of-care literature rather than from HEART score validation studies and are cited here only to characterize the off-hours operational environment. They are not evidence about the score itself: the weekend mortality effect operates through treatment access delays and reperfusion timing, whereas the present analysis concerns the discriminative accuracy of a score applied at initial triage, which these data do not address.

Pre-specified secondary analyses confirmed the primary finding of clinical similarity across multiple comparison frameworks. The Mantel-Haenszel pooled odds ratio for 30-day MACE, with HEART risk group as the stratification variable, was 0.989, indicating near-identical risk within each risk stratum regardless of shift assignment. Calibration metrics were statistically indistinguishable between shift groups, with a calibration intercept difference of -0.0003 (*p* = 0.998) and a slope difference of 0.070 (*p* = 0.426), confirming that systematic miscalibration relative to the reference cohort probabilities reflects population-level rather than shift-specific factors. Decision curves demonstrated positive net benefit over treat-all and treat-none strategies across the full threshold range of 5–40%, with a net advantage of 0.023 per patient at a threshold of 10% in the off-hours group. In a post-hoc exploratory analysis, the Youden-index optimal threshold was HEART ≥ 6, yielding sensitivity of 75.4% (95% CI 70.3–79.9%) and specificity of 77.5% (95% CI 75.5–79.4%); this threshold was not pre-specified and should be regarded as hypothesis-generating only.

Discrimination and calibration describe different and non-interchangeable properties of a risk score. Discrimination is the capacity to rank patients by relative risk, and it was preserved here, with an AUC near 0.83 in both shift groups. Calibration is the agreement between the absolute probabilities a model assigns, and the event frequencies actually observed, and it was systematically off: the reference-cohort probabilities overestimated absolute MACE risk across the score range (calibration intercept − 0.352; overall observed-to-expected ratio 0.81; slope 0.83). The Hosmer-Lemeshow test was significant, but this test loses specificity in large samples, where minor and clinically unimportant departures from perfect fit are routinely flagged [17]; the intercept and the observed-to-expected ratio are more informative descriptors of the direction and magnitude of miscalibration here. The clinical consequence depends on how the score is used. When the HEART score is applied as an ordinal triage tool, with rule-out at a fixed cut point such as HEART > = 4, only the ranking matters, and the present results support its use unchanged across all hours of presentation. When the same score is used to communicate or act on an absolute probability of MACE, the reference-cohort estimates would overstate a patient’s true risk in this population and could prompt unnecessary admission or testing. A model that discriminates well but is miscalibrated can therefore mislead probability-based decisions even when its ranking is sound [18]. This distinction, rather than any shift-related effect, is the practical limitation of applying published HEART probabilities locally.

These findings carry direct operational consequences for emergency department triage at all hours of presentation. At the rule-out threshold of HEART ≥ 4, the off-hours cohort showed a negative predictive value of 97.7%, with 13 missed events among 574 low-risk patients, a 2.3% miss rate consistent with the accepted bounds for structured early discharge protocols. A stepped-wedge cluster randomized trial of HEART score implementation across nine Dutch emergency departments found HEART-guided care to be safe, with six-week MACE incidence 1.3% lower in the HEART care group than in usual care, within that trial’s noninferiority margin [19]. The high-risk threshold (HEART ≥ 7) yielded a positive likelihood ratio of 6.07, indicating a clinically meaningful increase in post-test probability; this rule-in performance was preserved across all five-time strata. When the HEART pathway is combined with a 0-hour/1-hour high-sensitivity troponin protocol, NPV for 30-day MACE reaches 99.8%, identifying 49.8% of patients for safe rule-out without additional testing [20]. The AUC of 0.830 at a 14.4% MACE rate is consistent with discrimination expected at this prevalence level across published external validation datasets [14, 15], and the absence of a shift-related AUC difference confirms that off-hours operational constraints do not attenuate the score’s triage gradient. Across both shift groups, positive net clinical benefit was observed at all threshold probabilities between 5% and 40%, indicating that neither overtriage nor undertriage is differentially amplified by off-hours conditions.

The retrospective single-center design limits generalizability to tertiary academic emergency departments with comparable infrastructure; settings with more severe off-hours constraints or less standardized assay platforms may show different performance. Patients presenting with ST-elevation myocardial infarction were excluded by design, because they bypass HEART-based triage and proceed directly to reperfusion; these findings therefore apply to the undifferentiated chest pain population in which the HEART score is used and do not extend to the broader acute coronary syndrome spectrum that includes ST-elevation myocardial infarction. Serial troponin completion was 12.5% points lower during off-hours, and restriction to patients with complete second-troponin documentation raised the off-hours AUC from 0.830 to 0.841, indicating modest downward bias in the primary estimate. The MACE composite was restricted to unplanned coronary revascularization, but procedural urgency was ascertained from administrative records; residual misclassification of elective procedures, if present, would inflate the observed MACE rate and reduce rule-out performance metrics. A mid-study transition from conventional to high-sensitivity troponin I created temporal confounding between assay type and shift assignment; assay-stratified analyses produced overlapping confidence intervals across both platforms, suggesting limited influence on the primary AUC. Observer variability in the History and ECG components is inherent to retrospective HEART score abstraction, though the seniority subgroup analysis produced AUC values of 0.824, 0.833, and 0.827 across resident, specialist, and cardiologist groups, consistent with non-differential misclassification. Thirty-day outcomes were ascertained from institutional records without active patient contact; events occurring at external facilities would not be captured, biasing the observed event rate downward and the negative predictive value upward, with magnitude unquantifiable from available data. The off-hours definition encompassed evenings, nights, weekends, and public holidays, covering approximately 75% of weekly calendar hours and producing a marked imbalance in group sizes (off-hours 76.2%, on-hours 23.8%); this imbalance is a limitation, as the smaller on-hours group (*n* = 667) widened its AUC confidence interval (95% CI 0.778–0.865), although the margin was still met with adequate precision. To address this asymmetry, balanced alternative time definitions (weekday versus weekend; daytime versus nighttime) were examined post hoc; neither contrast showed a significant difference in discrimination (both *p* > 0.14) and both point estimates remained within the a priori margin, supporting the primary finding under more balanced groupings.

The 0.08 margin of clinical similarity was a pragmatic threshold set a priori rather than one derived from an external empirical benchmark, and a different margin could alter the interpretation; the descriptive AUC difference and its confidence interval should therefore be read alongside the margin rather than as a formal equivalence claim. The same sample size asymmetry reduces precision of the on-hours calibration summary estimate; the overall O/E ratio for on-hours (0.791; 95% CI 0.642–0.965) carries substantially wider uncertainty than the off-hours estimate (0.812; 95% CI 0.724–0.908), and between-group calibration comparisons should be interpreted with this limitation in mind.

These findings suggest that the HEART score may warrant prospective evaluation as a time-agnostic triage instrument in multicenter cohorts across diverse institutional and geographic settings. The stepped-wedge implementation trial confirmed that HEART-guided care is safe but identified physician nonadherence to low-risk discharge recommendations as the principal barrier to realizing its efficiency benefits [19], a challenge that may be amplified during off-hours when decision-making operates under greater time pressure. The systematic miscalibration identified relative to the reference-cohort probabilities in both shift groups suggests that local recalibration of absolute risk estimates should be considered before probability-guided triage protocols are implemented. Because this miscalibration signal derives from single-center retrospective data, the magnitude and direction of any recalibration should be confirmed prospectively rather than applied directly from these estimates. Recalibration of the HEART score troponin threshold to the limit of detection with high-sensitivity assays has been shown to increase sensitivity for rule-out from 96.1% to 98.6% while maintaining specificity, supporting the feasibility of this approach [21]. A prospective stepped-wedge or cluster-randomized implementation trial, incorporating standardized ECG adjudication by shift category and concurrent troponin assay documentation, is required to determine whether discrimination remains clinically similar across centers with different off-hours staffing models. Such a trial should also assess whether locally recalibrated probability thresholds improve absolute risk communication without compromising the safety of threshold-based rule-out.

## Conclusion

In adult emergency department patients presenting with acute chest pain, the HEART score demonstrated clinically similar discrimination for 30-day MACE regardless of presentation time, with an AUC of 0.830 (95% CI 0.805–0.855) in off-hours and 0.821 (95% CI 0.778–0.865) in on-hours presentations, both falling within the ± 0.08 margin of clinical similarity. These findings support the use of the HEART score across different presentation times, including evenings, nights, weekends, and public holidays, in similar tertiary-care settings, with preserved sensitivity and negative predictive value at the rule-out threshold.This applies to tertiary academic centers with comparable operational and assay infrastructure and requires prospective multicenter confirmation. Systematic overestimation of absolute MACE risk relative to the reference cohort probabilities was observed equally across shift groups indicating that local recalibration of predicted risk should be considered before probability-guided triage protocols are adopted, irrespective of presentation time.

Prospective multicenter validation across diverse emergency department settings, assay types, and shift staffing models is required before these findings can be incorporated into practice guidelines for time-stratified chest pain triage.

## Supplementary Information

Below is the link to the electronic supplementary material.

Supplementary Material 1

## Data Availability

The data that support the findings of this study are not publicly available due to patient privacy constraints and institutional ethics committee restrictions. De-identified aggregate data and the analysis code are available from the corresponding author upon reasonable request, subject to approval by the Istanbul Medipol University Non-Interventional Clinical Research Ethics Committee.

## Abbreviations

ACS: Acute coronary syndrome
AUC: Area under the receiver operating characteristic curve
CI: Confidence interval
DCA: Decision curve analysis
ECG: Electrocardiogram
EDACS: Emergency Department Assessment of Chest Pain Score
FDR: False discovery rate
GRACE: Global Registry of Acute Coronary Events score
HEART: History, Electrocardiogram, Age, Risk Factors, Troponin score
hsTnI: High-sensitivity troponin I
IQR: Interquartile range
LR: Likelihood ratio
MACE: Major adverse cardiac event
MICE: Multiple imputation by chained equations
NPV: Negative predictive value
NSTEACS: Non-ST-elevation acute coronary syndrome
O/E: Observed-to-expected ratio
OR: Odds ratio
PPV: Positive predictive value
ROC: Receiver operating characteristic curve
SA: Sensitivity analysis
SMD: Standardised mean difference
STEMI: ST-elevation myocardial infarction
TIMI: Thrombolysis in Myocardial Infarction score
TRIPOD: Transparent Reporting of a Multivariable Prediction Model for Individual Prognosis or Diagnosis
