# Supplementary material for: Time-stratified HEART score discrimination and calibration in emergency chest pain: a retrospective cohort study
Source: BMC Emerg Med. 2026 Jun 17;26:231. doi: 10.1186/s12873-026-01652-2 (PMC13520530; doi:10.1186/s12873-026-01652-2)
Supplement: Supplementary file 1 [file 12873_2026_1652_MOESM1_ESM.docx]

**Supplementary Documents**

**Supplementary Methods 1. Normality assessment protocol.**

For samples with n ≥ 50, normality was assessed using a combined multi-criterion protocol defined a priori in the analysis plan: a variable was classified as non-normal if at least three of five criteria indicated departure from normality, comprising the Kolmogorov-Smirnov test with Lilliefors correction (p ≤ 0.05), skewness z-score ≥ 1.96, kurtosis z-score ≥ 1.96, Q-Q plot visual inspection, and histogram shape assessment. No single published guideline mandates this specific combination of criteria. For samples with n < 50, the Shapiro-Wilk test was applied.

**Supplementary Table S1**. Sensitivity analyses: off-hours cohort performance across subgroups and alternative analysis strategies.

| **Sensitivity analysis** | **n** | **Events, n (%)** | **AUC (95% CI)** | **Calibration intercept** | **Brier** | **vs primary** |
| --- | --- | --- | --- | --- | --- | --- |
| **Primary (off-hours, full cohort)** | **2,133** | **305 (14.3)** | **0.830 (0.805-0.855)** | **-0.352** | **0.101** | **Ref.** |
| SA1: Original serial troponin complete | 1,681 | 243 (14.5) | 0.841 (0.815-0.867) | -0.294 | 0.098 | +0.011 |
| SA2a: hsTnI assay | 1,458 | 211 (14.5) | 0.821 (0.791-0.851) | -0.391 |  |  |
| SA2b: Conventional TnI assay | 675 | 94 (13.9) | 0.848 (0.804-0.892) | -0.263 |  |  |
| SA3: Resident-interpreted ECG | 663 | 103 (15.5) | 0.824 (0.781-0.867) | -0.335 |  |  |
| SA3: Specialist-interpreted ECG | 1,183 | 162 (13.7) | 0.833 (0.802-0.865) | -0.360 |  |  |
| SA3: Cardiologist-interpreted ECG | 287 | 40 (13.9) | 0.827 (0.743-0.911) | -0.361 |  |  |
| SA4: 6-week MACE endpoint | 2,133 | 332 (15.6) | 0.818 (0.793-0.843) | -0.211 |  | -0.012 |
| SA5: MICE-imputed (HEART_MICE) | 2,133 | 305 (14.3) | 0.835 (0.810-0.859) | -0.360 |  | +0.005 |
| Complete case (all secondary variables present) | 1,529 | 219 (14.3) | 0.837 (0.809-0.865) | -0.296 | 0.100 | +0.007 |

*All analyses restricted to off-hours group unless noted. SA = sensitivity analysis; hsTnI = high-sensitivity troponin I; MICE = multiple imputation by chained equations (20 imputation sets; 50 iterations; seed=2024). Calibration intercept estimated by offset logistic regression (H₀: intercept=0); Reference cohort risk-group probabilities used as fixed offset for all analyses.. All analyses except SA5 used original, non-imputed HEART scores; for SA5, predicted probabilities were derived from MICE-imputed HEART score risk-group assignments (20 imputation sets; 50 iterations; seed=2024; Rubin's rules applied). "vs primary" = difference in AUC point estimate fro*m primary analysis. Brier score on original reference cohort predicted probabilities where applicable.

**Supplementary Table S2**. AUC by time stratum with Cochran Q omnibus test results.

| **Time stratum** | **n** | **MACE events, n (%)** | **AUC (95% CI)** | **ΔAUC vs on-hours** | **FDR q** |
| --- | --- | --- | --- | --- | --- |
| On-hours (reference) | 667 | 97 (14.5) | 0.821 (0.778-0.865) | Ref. | Ref. |
| Evening | 450 | 71 (15.8) | 0.855 (0.810-0.899) | +0.034 | N/A |
| Night | 700 | 91 (13.0) | 0.840 (0.797-0.883) | +0.019 | N/A |
| Weekend daytime | 650 | 95 (14.6) | 0.799 (0.747-0.850) | -0.022 | N/A |
| Public holiday | 333 | 48 (14.4) | 0.835 (0.779-0.891) | +0.014 | N/A |

*Cochran Q statistic = 2.991, df=4, p=0.559. Global test is not significant; pairwise comparisons and FDR correction are not performed (pre-specified decision rule). AUC computed by DeLong method within each stratum independently. MACE = major adverse cardiac event; FDR = false discovery rate.*

**Supplementary Table S3.** 30-day MACE rates by HEART risk group and shift assignment.

| **Risk group** | **n** | **Off-hours MACE, n (%)** | **n** | **On-hours MACE, n (%)** | **p** |
| --- | --- | --- | --- | --- | --- |
| Low (HEART 0-3) | 574 | 13/574 (2.3) | 196 | 3/196 (1.5) | 0.740 |
| Intermediate (HEART 4-6) | 1,223 | 123/1,223 (10.1) | 360 | 43/360 (11.9) | 0.353 |
| High (HEART 7-10) | 336 | 169/336 (50.3) | 111 | 51/111 (45.9) | 0.493 |
| Mantel-Haenszel pooled OR (off-hours vs on-hours) | 2,800 | 0.989 (0.752-1.302) |  |  | 0.995 |

*Chi-square with continuity correction per risk stratum. Mantel-Haenszel pooled OR: off-hours vs on-hours, HEART risk group as stratification variable. CI estimated by Robins-Breslow-Greenland variance method. Global p-value from Cochran-Mantel-Haenszel chi-square test (df=1).*

**Supplementary Table S4.** HEART score discrimination under balanced alternative time definitions (post hoc).

| **Comparison** | **Group** | **n** | **Events, n (%)** | **AUC (95% CI)** | **ΔAUC (95% CI)** | **p** |
| --- | --- | --- | --- | --- | --- | --- |
| Weekday vs weekend (calendar) | Weekday (Mon-Fri) | 1,967 | 284 (14.4) | 0.839 (0.813-0.864) | – | – |
|  | Weekend (Sat/Sun) | 833 | 118 (14.2) | 0.803 (0.764-0.843) | weekend − weekday: -0.035 (-0.082 to +0.012) | 0.142 |
| Daytime vs nighttime (clock) | Daytime (08:00-17:59) | 1,256 | 182 (14.5) | 0.810 (0.776-0.844) | – | – |
|  | Nighttime (18:00-07:59) | 1,544 | 220 (14.2) | 0.843 (0.816-0.869) | daytime − nighttime: -0.032 (-0.076 to +0.011) | 0.145 |

*Post hoc, exploratory analyses examining HEART score discrimination under balanced alternative time definitions, added to address the asymmetry of the off-hours definition. AUC by the DeLong method; ΔAUC compared between independent groups using DeLong variance. Weekday/weekend defined by calendar day; daytime/nighttime defined by triage hour. Both point estimates fall within the a priori margin of 0.08. Abbreviations as in Tables 1-2.*

**Supplementary Figure S1.** *Flow of participants through the study. Patients presenting with acute non-traumatic chest pain were screened consecutively from January 2020 through December 2025. Off-hours encompassed evenings (18:00-23:59), nights (00:00-07:59), weekends, and public holidays; on-hours encompassed regular weekday working hours (08:00-17:59). MACE = major adverse cardiac event (composite of acute myocardial infarction, unplanned coronary revascularization, cardiac arrest, or all-cause death within 30 days); dx = diagnosis.*

**Supplementary Figure S2.** *Left: 30-day MACE component rates by HEART risk group. Right: 30-day MACE event rate by time stratum with sample sizes. Abbreviations: AMI = acute myocardial infarction; MACE = major adverse cardiac event.*

**Supplementary Figure S3.** Forest plot of sensitivity analyses. Primary analysis shown in dark blue; sensitivity analyses in lighter shades. Error bars represent DeLong 95% confidence intervals. The dashed vertical line marks the primary AUC estimate (0.830). All sensitivity analyses produced AUC estimates within the clinical-similarity margin. SA = sensitivity analysis; MICE = multiple imputation by chained equations; hsTnI = high-sensitivity troponin I.
